# Exploitation of pig by-product: physicochemical properties and flavor of Dachang Tang: effect of cooking time

**DOI:** 10.5713/ab.24.0894

**Published:** 2025-04-28

**Authors:** Tongxiang Yang, Jiayin Wei, Xiangtian Wu, Yunfei Wang, Kongyang Wu, Xu Duan, Wenchao Liu, Weiwei Cao, Linlin Li, Chung Lim Law, Junliang Chen, Guangyue Ren

**Affiliations:** 1 College of Food and Bioengineering, Henan University of Science and Technology, Luoyang, China; 2 Henan Province Engineering Research Center of Agricultural Products Processing Equipment, Luoyang, China; 3 Henan Province Engineering Technology Research Center of Agricultural Product Drying Equipment, Luoyang, China; 4 College of Life Science, Luoyang Normal University, Luoyang, China; 5 Department of Chemical and Process Engineering, Malaysia Campus, University of Nottingham, Semenyih, Malaysia

**Keywords:** Cooking Time, Microstructure, Physicochemical Properties, Pig Large Intestine, Sensory, Volatile Compounds

## Abstract

**Objective:**

The objective of this study was to determine the optimal cooking time for Dachang Tang in order to promote Chinese cuisine culture and to improve the utilization of pig large intestines, thereby reducing resource waste.

**Methods:**

The raw pig large intestines were cleaned with water and flour, then boiled with selected spices for 40 mins to reduce off-odors. Subsequently, the large intestines were stir-fried with oil, garlic, and *Zanthoxylum bungeanum* for a specified duration, before being added to the original broth. The pig large intestines were then cooked for 10 mins, 20 mins, 40 mins, and 60 mins, respectively. Samples were collected at each cooking times to analyze their basic nutritional components (moisture, protein, fat), texture, color, water state, fatty acids, volatile compounds, microstructure, and sensory characteristics.

**Results:**

With the increase in cooking time, the moisture and fat content decreased significantly, and the ratio of unsaturated to saturated fatty acids increased (p<0.05). At 20 mins, pig large intestines exhibited optimal chewiness, color, surface integrity and sensory scores. Meanwhile, volatile flavor compounds mainly derived from spices, such as 4-methoxybenzaldehyde, benzaldehyde, 3-ethyltoluene, estragole, and ethyl decanoate effectively masking the off-flavor caused by p-cresol and indole in pig large intestines, and provided fruity, spicy, fennel and citrus aromas to Dachang Tang.

**Conclusion:**

A comprehensive analysis concluded that 20 mins was the optimal cooking time.

## INTRODUCTION

Pork accounts for 33% of global meat consumption, with Asia contributing 56% of global pork production and China alone responsible for 48% [1]. The production of pig by-products during slaughter and processing leads to substantial resource waste because of low utilization, even though pork consumption is considerable. The pig large intestine (PLI) is one of the pork by-products, containing protein, fat content, as well as rich vitamins (especially B vitamins) and minerals (such as manganese, calcium, selenium, etc.) [2]. It’s also have been reported to be a valuable source of collagen, renowned for their cosmetic benefits, including maintaining skin elasticity [3]. Even so, the PLI still suffers from high production but low utilization rates. By converting PLI into a range of cuisines, this issue can be partially resolved. Developing PLI products can not only remove or reduce its odor but also make it a more widely accepted ingredient. This not only helps increase the market value of PLI but also reduces resource waste, promoting its efficient utilization.

There are many cuisines about PLI in China, such as “marinated large intestines”, “fried pork intestines”, the famous Shandong dish “red braised intestines”, and Anyang’s “fried blood intestines” from Henan. Dachang Tang (DCT) is an intangible cultural heritage delicacy from Henan Province, China, with PLI as the main ingredient, complemented by tofu and blood, and cooked with aromatic spices. It is highly popular among consumers. Cooking time is a key processing step during the cooking process [4,5]. Related studies have shown that increased cooking time reduces key odor-causing compounds, thereby mitigating unpleasant odors. However, cooking too long can also deteriorate the texture of the large intestines [3,6]. Li et al [6] found that continuously boiling PLI for 10 to 20 mins significantly reduces key odor-causing substances such as cresol and indole. Kim et al [3] identified 120 mins as the optimal cooking duration for odor removal, minimizing cooking loss, and achieving optimal shear force in PLI. Therefore, it is crucial to determine the cooking time during the processing of DCT.

The purpose of this study was to explore the effects of different cooking times on the fundamental nutritional components, color, texture, and microstructure of gourmet DCT and to explore the changes in its flavor by solid-phase microextraction gas chromatography-mass spectrometer (SPME-GC-MS). Through a comprehensive analysis, the study seeks to determine the optimal cooking time and provide theoretical insights for enhancing the texture and flavor of PLI. The findings aim to promote the utilization of pig by-products and support the industrial development of the DCT.

## MATERIALS AND METHODS

### Materials

In this study, PLI, seasonings and spices were purchased from Luoyang Dazhang Supermarket, with the PLI stored at −25°C in the refrigerator for later use.

### Preparation of sample

#### Raw Pig Large Intestine

PLI were removed from the refrigerator to thaw and washed with flour and tap water for further analysis.

**Figure 1:**
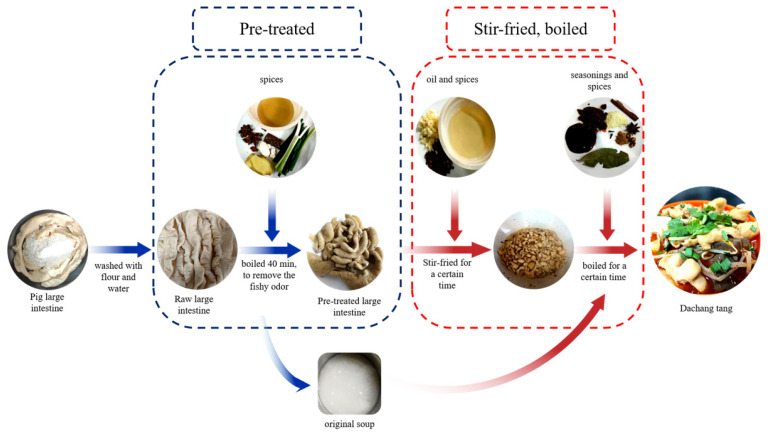
Processing diagram of Dachang Tang.

Figure 1 shows the processing of DCT. Pre-treated PLI (0 min): The cleaned thoroughly RL was blanched in boiling water. Then 4,000 g water and 200 g PLI were added to the pan with measured quantitative spices (scallion, ginger, star anise, cinnamon, angelica, and cooking wine), and were cooked at 900 W for 40 mins to reduce the off-odor of the PLIs. The pre-treated PLI and original soup were stored separately for further processing.

#### Cooked large intestine group (10–60 mins)

The pre-treated PLI was added to the pan with the garlic and *Zanthoxylum bungeanum*. Then, 1,600 g of the original soup was added to the pan, along with measured amounts of seasonings and spices (star anise, myrcia, cloves, cinnamon, and chili oil). When the soup began to simmer cooked at 900 W for 10 mins, 20 mins, 40 mins, and 60 mins, respectively. Samples of PLI were cut into different sizes to measure various indicators.

### Moisture, fat and protein analysis

The moisture, protein, and fat content were analyzed according to the method of AOAC (2006) and were followed precisely during analysis. The moisture content was determined using AOAC 950.46, which involved drying in an oven at 105°C. Fat content was determined using a fat analyzer (SOX500; Shandong Haineng Scientific Instrument, Dezhou, China), referring to AOAC method 991.36; Protein content was determined using a Kjeldahl nitrogen determinator (K1305A; Shanghai Shengsheng Automated Analytical Instrument, Shanghai, China) to determine the nitrogen content, which was converted to protein content by the formula N×6.25 (where N is the nitrogen content from the sample and 6.25 is the conversion factor), as described in AOAC 928.08. Analyses were performed in triplicate.

### Texture profile analysis

PLI was cut into unfolded lengths of about 120 mm×120 mm. The texture of the PLI was measured according to the method of Li et al [7] with slightly modified, by using a texture analyzer (TA. XT EXPRESS; SMS, Glasgow, UK). The measured parameters were as follows: strain, 70%; pretest speed, 1.0 mm/s; test speed, 2.0 mm/s; posttest speed, 5.0 mm/s; trigger force, 5.0 g; and time, 5.0 s. The hardness, springiness, cohesiveness, and chewiness were determined. Each sample was repeated five times to obtain an average value.

### Color analysis

PLI was cut into approximately 120 mm×120 mm lengths when unfolded. The colorimeter (Color i5; X-Rite, Grand Rapids, MI, USA) was used to measure the color of the samples. The color values were expressed as L* (lightness), a* (red-green value), and b* (yellow-blue value). Each sample was repeated three times to obtain an average value.

### Low-field nuclear magnetic resonance measurements

Low-field nuclear magnetic resonance (LF-NMR) relaxation was determined using a LF-NMR analyzer (NMI20-015V-I; Shanghai Neumay Electronic Technology, Shanghai, China). According to Wang et al [8] with slight modifications on the methodology. Approximately 1 g of the sample was placed into a cylindrical glass tube and inserted into the center of the magnetic field. The Carr-Purcell-Meiboom-Gill (CPMG) multiple-pulse echo sequence was used to collect the transverse relaxation time (T2). The relevant parameters were as follows: the time waiting = 4,000 ms, the time echo = 0.25 ms, the number of echoes = 3,000, and the number of scans = 4. Each sample was repeated three times to obtain an average value.

### Fatty acid analysis

Fatty acid (FAA) was extracted following the method of Folch et al [9]. 5 g of the sample was added to a mixture of 100 mL dichloromethane-methanol (2:1, V/V). The mixture was subjected to ultrasonication for 10 mins and 2 mL of saturated sodium chloride solution to facilitate phase separation. The lower layer was collected as the chloroform-methanol extract. Water was removed using anhydrous cupric sulfate, and the lipid sample was obtained by evaporating the solvent using the rotary evaporator at 50°C under vacuum.

FAA methyl esterification concerned Indrasti [10] and brief descriptions were as follows: 50 mg of extracted fat was placed in a test tube, 2 mL of benzene-petroleum ether mixture (1:1, V/V) was added, and mixed well, then 2 mL of 0.4 mol/mL KOH-CH_3_OH was added and mixed well. After leaving to stratify, saturated NaCl solution was added along the wall of the test tube to raise the organic phase layer. After clarification, the supernatant was taken and passed through a 0.22 μm filter membrane. The filtrate was loaded into a sample bottle for testing.

The composition of FAAs was determined by gas chromatography-mass spectrometry (GC-MS). The SP-5 2560 capillary column was used, the inlet temperature was 250°C, He was used as carrier gas, and the flow rate was 1.0 mL/min. The injection volume was 1 μL and the split ratio was 24: 1. Temperature programming: The initial temperature of the oven was set to 140°C for 2 mins, increased to 200°C at 6 °C/min for 6 mins, then increased to 230°C at 2°C/min for 2 mins, and finally reached to 250°C at 4°C/min for 2 mins. Conditional parameters: The transmission line temperature of the mass spectrometer was 250°C, and the temperature of the ion source was 230°C. The masses were scanned over a range of 40−400 m/z and a solvent delay of 10 mins. The qualitative analysis of FAAs was conducted using mass spectrometry library matching, while the quantification was performed using peak area normalization.

### Volatile substance analysis

#### Solid-phase microextraction gas chromatography-mass spectrometer analysis

The detection of volatile flavor substances by GC-MS. HS-SPME condition: The SPME fiber was aged at 250°C for 5 mins. 2.0 g of PLI and 2.0 mL of saturated sodium chloride solution were added to a 20 mL headspace vial. 1 μL of 0.816 g/mL 2-methyl-3-heptanone solution as an internal standard was added to the headspace vial. SPME fiber was inserted into the extraction needle and allowed to adsorb at 60°C for 30 mins, followed by desorption at 250°C for 5 mins.

#### GC condition

Chromatographic column HP-5 (30 m×0.32 mm, 0.25 μm); carrier gas was He with a flow rate of 1.0 mL/min. The injection port temperature was set to 250°C. The initial column temperature was 50°C, held for 2 mins, then increased at a rate of 5°C/min to 180°C, held for 5 mins, followed by a ramp of 10°C/min to 250°C, held for 5 mins. The injection was performed in splitless mode.

#### MS condition

The ion source used 70 eV electron impact ionization at 230°C. The scanning mass range was 35–500 m/z with a quadrupole temperature of 150°C and a transmission line temperature of 280°C. The peak areas of each volatile compound were compared with the peak area of the internal standard, and the calculations were according to formula (1) [11].


(1) $$C=\frac{A_{x}\times C_{0}\times V\times 1,000}{A_{0}\times m}$$

Where: *C* is the concentration of the volatile compound (mg/kg); *C_0_* is the concentration of the internal standard (mg/mL); *A_x_* is the peak area of the volatile compound; *A_0_* is the peak area of the internal standard; *V* is the volume of the internal standard added; *m* is the mass of the sample (kg).

#### Odor activity value analysis

odor activity value (OAV) is typically used to assess the impact of individual volatile compounds on the overall aroma. According to the method by Zhang et al [12], the OAV was calculated using formula (2):


(2) $$OAV=\frac{C}{T}$$

Where: *C* is the concentration of the volatile compound (mg/kg); *T* is the sensory threshold of the substance in water (mg/kg).

### Scanning electron microscopy analysis

The PLI was cut into unfolded lengths of about 5 mm×5 mm for scanning electron microscope (S4800; Hitachi, Tokyo, Japan) analysis. Dry the samples using a vacuum freeze dryer for 24 hours. Once dried, coated the samples with a thin layer of gold using a sputter coater, and transferred into an scanning electron microscopy (SEM) chamber for observation.

### Sensory analysis

The sensory panel consists of 15 females and 15 males, volunteers from college of food and bioengineering aged between 22 and 30 years old, who conducted sensory evaluations on the appearance, spice flavor, large intestine flavor, tenderness, and taste of the DCT. Each member rated each attribute of the sample on a scale of 1 to 20. The sensory scores table is shown in Table 1. The study was reviewed and approved by the Henan University of Science and Technology and informed consent was obtained from each subject prior to their participation in this study.

### Statistical analysis

The volatile compounds were processed using the National Institute of Standards and Technology (NIST - 11) standard substance mass spectrometry database, as well as the retention times of C7 and C40 n-alkane standards. Data were processed using Excel software, with three parallel experiments conducted, and results were represented as the mean value± standard deviation. Graphs were plotted using Origin 2024 software (OriginLab, Northampton, MA, USA). Significance analysis was performed using SPSS 26.0 software (IBM, Armonk, NY, USA), where p<0.05 indicates significant differences. Principal component analysis (PCA) was conducted using SIMIA 14.1.

## RESULTS AND DISCUSSION

### The effect of cooking time on protein, fat, and moisture content

**Figure 2:**
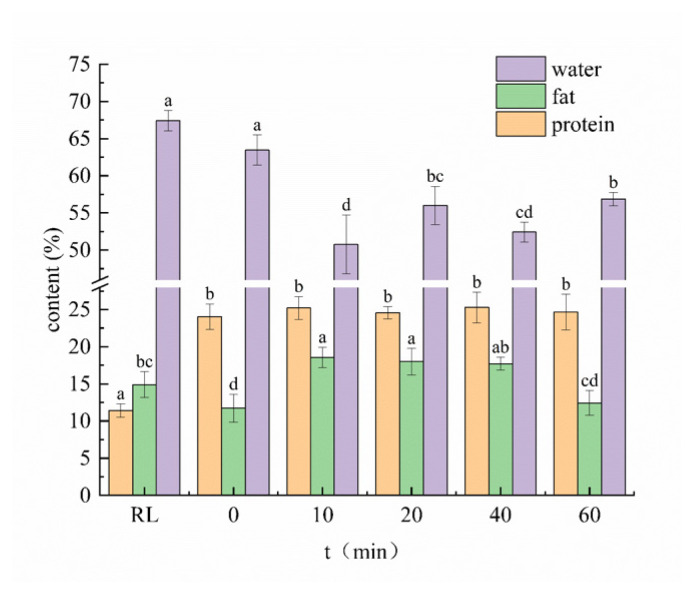
The effect of different cooking time on the water, fat, and protein content of RL and cooked PLI. Each values represents the mean±SD, n = 3. RL, raw pig large intestine; 0, pre-treated pig large intestine; 10, 20, 40 and 60 represent pre-treated pig large intestine cooked for 10 mins, 20 mins, 40 mins and 60 mins. ^a–d^ Different letters indicate significant differences between mean values for different cooking (p<0.05). PLI, pig large intestine; SD, standard deviation.

The results of the changes in the basic composition of the PLI under different cooking times are shown in Figure 2. During the cooking, the moisture content of PLI first decreased and then increased. It reached a minimum value of 50.75% at 10 mins, down from an initial value of 67.43%. This phenomenon can be attributed to the increased temperature in the center of the PLI during cooking, which induces protein denaturation and contraction, leading to a reduction in the water-holding capacity of the muscle fibers. This finding aligns with the study by Wang et al [13], which explored the impact of frying on pork tenderloin quality. Their study found that prolonged frying time at a constant temperature resulted in a reduction in the moisture content of the meat. The moisture content increased from 10 mins to 60 mins, reaching a peak value of 56.86% at 60 mins. This increase can be attributed to the extended cooking time, which facilitates the hydrolysis of collagen and myofibrillar proteins in PLI [14] thereby enhancing the penetration and absorption of water into the DCT. The RL exhibited the lowest protein content, which significantly increased after cooking. This increase may be attributed to the influence of nitrogen-containing substances present in the added spices.

Compared with RL, the fat content of PLI decreased significantly at 0 min (p<0.05), which was due to the formation of small molecules by the thermal reaction of fat during heating. These small molecules were dissolved in free water and flowed into the soup [15]. As the cooking time was extended, the fat content initially increased before subsequently decreasing. The fat content was highest during the 10−40 mins period, as this was when edible oil and chili oil were added during cooking. However, there was no significant difference in fat content between 60 mins and 0 min (p<0.05), indicating that the fat content in the large intestine was actually decreased during the cooking process. This reduction in fat content was primarily attributed to pre-frying, a finding that is in agreement with the changes in fat content observed after pre-frying and stewing of pork belly, as discussed by Li et al [16]. The mechanism needs further discussion. Comprehensive analysis indicated that at 20 mins, the moisture, fat, and protein content of PLI were all closest to those at 0 min.

### Changes in the texture during cooking

The RL exhibits the highest hardness and springiness (Table 2). Compared with RL, the hardness (g), springiness (mm), and chewiness (g·mm) of the large intestine decreased at 0 min, mainly because a large amount of collagen in PLI was degraded into gelatin, which tenderized the connective tissue. The results were consistent with the results of Li et al [6] exploring the effect of boiled time on the quality of PLI. From 0 to 60 mins, the hardness and springiness values initially increased and then decreased (p<0.05). At 20 mins, the hardness, springiness, and chewiness reached their maximum values. This was due to the cooking process, where the PLI underwent frying and boiling, causing an increase in the center temperature, loss of moisture, and denaturation of structural proteins. As a result, the surface of the large intestine shrank, forming a hard outer layer, which led to an increase in hardness [17,18]. 20–60 mins, collagen was converted into gelatin, which softened the connective tissue. This transformation was accompanied by a continuous decrease in myofibrillar protein content, along with the atrophy and breakage of muscle fibers, resulting in reduced hardness, elasticity, and chewiness. At 20 mins and 40 mins, the PLI exhibited higher springiness and chewiness, and which taste was better needed further sensory evaluation.

Cooking could cause muscle fibers to aggregate, leading to the stretching and dissociation of myofibrillar protein molecules [19]. This process accelerated the formation of disulfide bonds, resulting in moisture loss, increased toughness, and greater shear force in the meat. Conversely, boiling caused collagen dissolution, softened meat and reduced shear force [6,20]. Therefore, the shear force of meat during cooking depended on which of these two processes — aggregation of muscle fibers or gelatinization of collagen — was dominant.

### The influence of cooking time on the color

Color results are shown in Table 3. As cooking time increased, moisture gradually migrated from the interior to the surface in droplet form [21], leading to a significant decrease in L^*^ (brightness) (p<0.05). Both a* (redness) and b* (yellowness) initially increased and then decreased, reaching their maximum values at 20 mins. This change was attributed to high-temperature stir-frying and stewing, which induced Maillard reactions and fat oxidation, as well as the addition of seasonings, resulting in a deepening of color.

### Moisture status at different cooking times

A shorter relaxation time indicated a tighter bond between water and the substrate, reflecting lower water mobility, whereas a longer relaxation time signified higher water mobility [22]. 0−10 mins, the relaxation time significantly shortened (p<0.05), the relative content of immobile water (P21) decreased, and the content of free water (P22) increased (Table 4). The high heating rate during stir-frying caused muscle fibers to contract, expelling water from within the fibers into the extracellular space and converting the interstitial water into free water [23]. From 10 to 60 mins, the relaxation time was extended, and the P21 significantly increased (p<0.05). This was due to the conversion of collagen into gelatin in the PLI, which absorbed free water and compensated for the water loss in the muscle. Simultaneously, free water permeated the surface of the PLI and was lost.

### Effect of cooking time on fatty acids

The FAA results are shown in Table 5. The relative percentage content of FAAs reflects the composition ratio of each FAA at the same sampling point. Due to the continuous decrease in fat content during the cooking, the actual content of each FAA in PLI also decreased (Figure 2), in proportion to the reduction in fat content.

The FAAs in PLI included saturated fatty acid (SFA), monounsaturated fatty acid (MUFA), and polyunsaturated fatty acid (PUFA). C22:0 was identified in cooked PLI, but was not detected in either RL or pre-treated PLI. It may have originated from the spices, cooking oil, or chili oil added during the cooking process. Among the SFAs, C16:0 and C18:0 are the primary FAAs. As the cooking time increased from 10 to 60 mins, the content of SFAs significantly decreased (p< 0.05), falling from 51.56% to 44.29%, with the lowest value at 60 mins. The decrease in SFA content can be attributed to two main factors: the degradation of SFAs into low molecular weight compounds and polymers, and the transfer of SFAs from the meat into the broth. C18:1n9c was the primary MUFA and had a significant impact on the changes in MUFA content, with its relative content reaching the lowest at 60 mins. C18:2n6c was the primary PUFA, its trend was consistent with that of UFAs, showing a significant increase in relative content with longer cooking times (p<0.05), peaking at 60 mins. The increase in UFA was attributed to the generation of highly active antioxidants, including melanoidin-related compounds, which can eliminate hydroxyl radicals, superoxide radicals, and peroxides. These components inhibited the auto-oxidation of UFA, thereby preventing their oxidation and degradation during prolonged cooking. Within a certain range, a higher ratio UFA/SFA in food was beneficial, as UFAs helped prevent conditions such as arteriosclerosis and cardiovascular diseases, as well as contributed to the reduction of low-density lipoprotein cholesterol.

### Analysis of flavor compounds

A total of 56 substances were detected, broadly classified into 10 categories, including 10 ketones, 8 aldehydes, 9 alcohols, 2 phenols, 4 ethers, 9 esters, 1 acid, 4 hydrocarbons, 4 aromatics, and 5 other compounds (Supplement 1). There were 23 flavor compounds in RL, and the number and content of volatile compounds in PLI increased after cooking, and reaching a maximum at 20 mins. At this time, the number of compounds increased from 23 to 32, and the content increased from 111.54±5.21 to 194.73±18.84 mg/kg. The main volatile compounds in RL were ketones, and aldehydes and ethers increased significantly after cooking. This may be due to the oxidation and degradation of fats and proteins in PLI during processes like frying and boiling [24], as well as the addition of spices, which enriched the variety and content of flavor compounds. Some compounds mainly derived from spices such as (*Z*)-cinnamaldehyde, 4-methoxybenzaldehyde, eugenol, etc. reached a maximum value at 20 mins of cooking. At this time, substances representing the fecal odor of PLI, such as *p*-cresol, indole, and 3-methylindole, were not detected. It showed that cooking can not only enrich the flavor of DCT but also cover up or eliminate the unpleasant flavor. Interestingly, no fecal odor substances were detected at 20 mins and 60 mins, and a small amount of fecal odor substances were detected at 40 mins. The mechanism needs to be further explored.

During the cooking process, ketones were the most abundant volatile compounds in PLI, primarily produced through fat oxidation, Maillard reactions, and amino acid degradation [25]. Key volatile compounds in PLI included 3-heptanone, 2, 4-dimethyl-3-heptanone, and 4’-methylacetophenone, which contributed floral, fruity, and green aromas. Butyrophenone, with its camphor-like odor, may be a source of off-flavor in PLI and warrants further investigation. The absence of 2-pentadecanone in RL, suggests it might be derived from the added spices, contributing floral and fatty aromas.

Aldehydes were not found in RL. After cooking, their content was second only to that of ketones, increased from 27.61± 2.13 mg/kg to 55.46±5.28 mg/kg, indicating that aldehydes mainly originated from fat oxidation and the added spices. For example, benzaldehyde and nonanal, which derived from fat oxidation, primarily contribute to almond and fatty aromas [26,27]. (*Z*)-Cinnamaldehyde, 2-methoxybenzaldehyde, and 4-methoxybenzaldehyde primarily originated from star anise and cinnamon, imparting spicy, cinnamon, anise, and hawthorn flavors. Notably, (*Z*)-cinnamaldehyde, the most abundant aldehyde, served as a key characteristic flavor compound. Aldehydes typically have low odor thresholds, meaning they can impart distinctive aromas even in small amounts [28].

In RL, the main alcohols detected were 2,3-dimethylpentane-2-ol and phenylethanol, and the content decreased significantly after processing, reaching the lowest at 60 mins. It indicated that both compounds may have originated from RL. Eucalyptol, linalool, 4-terpineol, *α*-terpineol, and cinnamyl alcohol were only found in cooked intestines, mainly primarily came from spices such as oil, ginger, and star anise used during cooking [29,30], imparting herbal, spicy, woody, cinnamon, citrus, and rose odors to the DCT, suggesting that alcohols are a major contributor to flavor in spices.

*p*-Cresol, indole, and 3-methylindole were the primary compounds responsible for the off-flavors in PLI [6], originating mainly from the degradation of tryptophan and tyrosine [31]. These compounds were correlated with fecal and horsey odors commonly found in fermentation foods [6]. After cooking, the concentration of these off-odor compounds significantly decreased or disappeared, indicating that boiling can reduce or mask unpleasant flavors to some extent. Eugenol, present in small amounts in RL, mainly came from added spices such as cloves and peppers, providing clove and spicy flavors. Anethole, the most abundant flavor compound, offered licorice and anise-like aromas and was present in small amounts in RL, primarily derived from added spices such as star anise.

Esters, typically formed through the esterification of acids and alcohols, are characterized by sweet and fruity flavors [32]. As cooking time increased, the content rose from 2.09± 0.49 mg/kg to 10.80±2.41 mg/kg, reaching a peak at 60 mins. Hydrocarbons and aromatic compounds, due to their low content and high threshold values, played a minor role in the overall flavor.

### Odor activity value analysis

The perception of flavor ultimately depended on both the concentration and threshold value of the compounds. Typically, OAV of 1 or greater was considered to have a significant impact on flavor; the larger the OAV, the greater the contribution to the overall flavor [33]. The results further confirmed that the primary sources of off-flavors in RL were *p*-cresol, indole, and 3-methylindole (OAV>50) (Table 6), and these off-odor substances were not detected at 20 mins of cooking. 3-Heptanone and 5-nonanone were the primary compounds providing fatty and floral aromas in RL and the main contributors to the persistent fatty aroma throughout the cooking process. As the cooking time extended (10–60 mins), the flavor compounds from the spices increased and gradually reached equilibrium. At 20 mins, the volatile compounds were abundant, with some reaching their maximum flavor contribution, including 5-nonanone, benzaldehyde, 4-methoxybenzaldehyde, estragole, ethyl decanoate, and phellandrene, which provide almond, cherry, anise, spicy, woody, and fruity flavors. However, some compounds have not yet fully volatilized at this stage. By 60 mins, substances with OAV values greater than 1 were as abundant as at 20 mins, but the flavor compounds were different. The flavor compounds included eucalyptol, linalool, eugenol, and anethole, which provide anise, herbal, floral, citrus, rose, spicy, and licorice flavors. These compounds effectively mask the fecal odors of PLI and enhance the overall aroma of the cooked PLI.

### Partial least squares discriminant analysis

**Figure 3:**
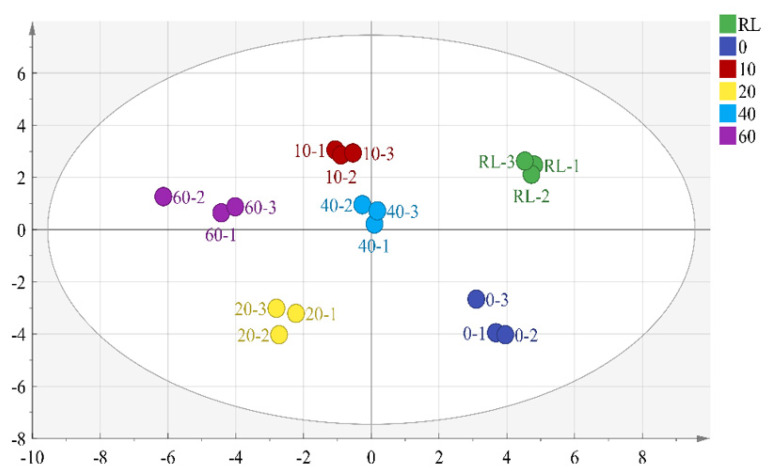
Partial least squares discriminant analysis (PLS-DA) of volatile substances in RL and cooked PLI at different cooking time. RL, raw pig large intestine; 0, pre-treated pig large intestine; 10, 20, 40 and 60 represent pre-treated pig large intestine cooked for 10 mins, 20 mins, 40 mins and 60 mins. PLI, pig large intestine.

**Figure 4:**
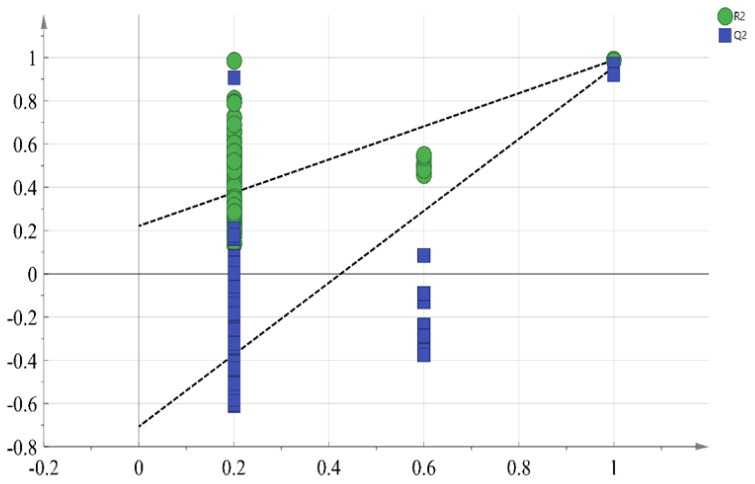
Permutation plot for PLS-DA model. R2, explained variance. Q2, predictive ability. PLS-DA, partial least squares discriminant analysis.

Partial least squares discriminant analysis (PLS-DA) is a supervised PCA method that performs dimensionality reduction while incorporating group information. This multivariate statistical technique models regression on multiple independent variables and accurately identifies the most important variables influencing the grouping. Therefore, PLS-DA is effective for further screening of the most crucial flavor compounds [34]. The compounds with OAV values greater than 0.1 were used as independent variables, RL and PLI cooked at different times (0–60 mins) as dependent variables. Figure 3 showed the PLS-DA plot, where RL and pre-treated PLI were located on the positive axis of PC1, while cooked PLI were located on the negative axis of PC1. Cooked PLI at 20 mins and 60 mins were the furthest from RL, indicating the greatest difference. This distinction arises because, during the cooking process, the volatile compounds from spices and the flavor compounds formed from fat oxidation gradually reach a dynamic equilibrium. The PLS-DA prediction model, obtained through interaction residual variance analysis (Figure 4), has an explanatory variable R2X of 0.939, R2Y of 0.982, and a predictive ability Q2 of 0.948. These values indicated that the model is effective and not overfitted, suggesting that the results can be used for this sample analysis [35].

**Figure 5:**
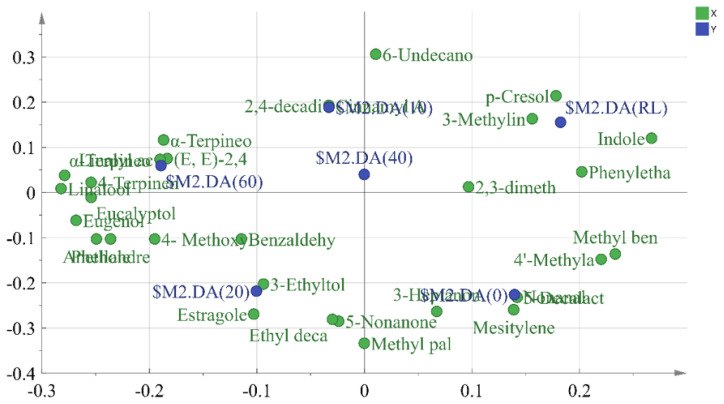
Loading scatter plot for PLS-DA model. RL, raw pig large intestine; 0, pre-treated pig large intestine; 10, 20, 40 and 60 represent pre-treated pig large intestine cooked for 10 mins, 20 mins, 40 mins and 60 mins. PLS-DA, partial least squares discriminant analysis.

Loading scatter plot (Figure 5) from the PLS-DA analysis were used to assess the contribution of flavor substances to RL and various cooking durations. The figure revealed that the off-flavors in RL mainly originate from p-cresol, indole, and 3-methylindole. At 0 min, the main flavor compounds in PLI were nonanal, mesitylene, 5-decalactone, 4’-methylacetophenone, and methyl benzoate, which primarily providing fatty aroma. As the cooking time increased, aromatic compounds from the spices volatilized and became important flavor contributors. At 20 mins, the compounds with the greatest contribution were 4-methoxybenzaldehyde, benzaldehyde, 3-ethyltoluene, estragole, and ethyl decanoate, providing fruity, spicy, anise, and citrus flavors. At 60 mins, the characteristic flavor compounds were *α*-terpineol, linalyl acetate, (*E, E*)-2, 4-decadienal, *α*-terpineol acetate, and 4-terpineol. These compounds, primarily derived from alcohols and esters, imparting woody, citrus, herbal, spicy, and fatty flavors.

### Analysis of microscopic structure

**Figure 6:**
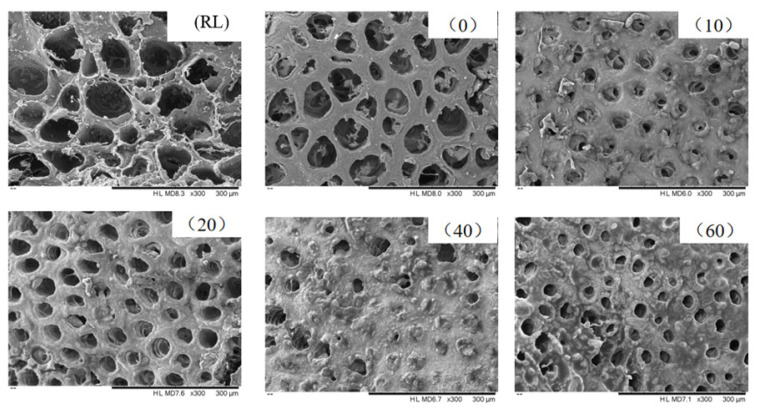
The effect of different cooking times on the microstructure of RL and cooked PLI (bar = 300 μm). RL, raw pig large intestine; 0, pre-treated pig large intestine; 10, 20, 40 and 60 represent pre-treated pig large intestine cooked for 10 mins, 20 mins, 40 mins and 60 mins. PLI, pig large intestine.

The SEM results are shown in Figure 6. RL exhibited larger pores and a rough surface. During the pre-treatment, the heating caused the PLI to contract, which led to the absorption of moisture by the muscle cells, resulting in a smoother surface and a reduction in pore size. From 0 to 10 mins, the rapid increase in temperature due to frying and boiling led to the surface of the PLI to contract and a decrease in pore size. The main reason for this was the conversion of immobile water in PLI to free water and the loss of some of the free water (Table 5). The primary process during cooking involved the dynamic exchange of fat and moisture between PLI and the broth (Figure 2). The exchange of moisture involved the denaturation and contraction of proteins in PLI, reducing its water-holding capacity. Additionally, the hydrolysis of collagen and myofibrils facilitated the absorption of moisture from the broth. The adhesion of oil from the broth (such as added chili oil and cooking oil) to the PLI’s surface, combined with the breakdown and dissolution of fat cells into the broth, results in a dynamic exchange of fat. At 20 mins, the surface of PLI appears relatively smooth with clear pores. The texture results also indicated the highest levels of springiness and chewiness (Table 2). This may be the optimal cooking time and needs to be further verified by sensory evaluation.

### Sensory analysis

**Figure 7:**
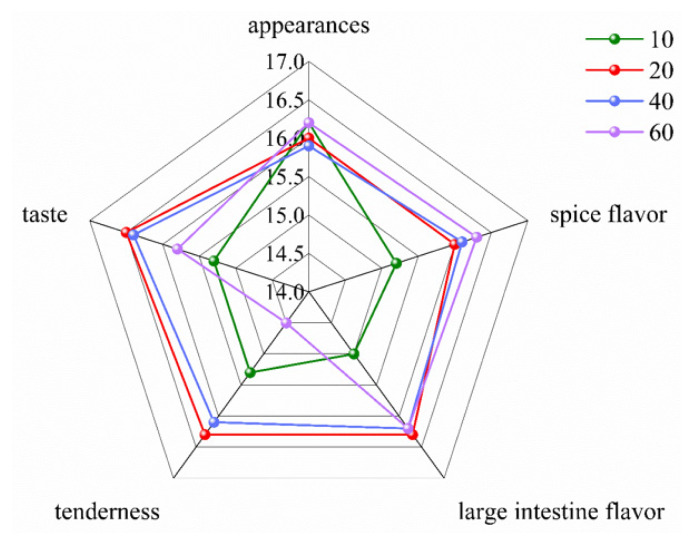
The sensory evaluation of cooked PLI at different cooking times. 10, 20, 40 and 60 represent pre-treated pig large intestine cooked for 10 mins, 20 mins, 40 mins and 60 mins. PLI, pig large intestine.

The results of sensory analysis of cooked large intestines are shown in Figure 7. Cooked PLI showed no significant differences in appearance. The spice aroma score was lowest at 10 mins because the short cooking time did not allow for the complete volatilization of flavor compounds. 20–60 mins, as the cooking time extended, the flavor compounds gradually volatilize. Both spice aroma and PLI flavors scores were relatively high, resulting in a rich fragrance. The tenderness and taste of the intestines were optimal at 20 mins and 40 mins, when PLI had good elasticity, were easy to chew, and had a balanced salty and spicy flavor, providing the best mouthfeel. At 10 mins, the relatively short cooking time resulted in poor chewiness (Table 2) and incomplete penetration of the seasonings into the intestines, making the flavor somewhat bland. At 60 mins, the longer cooking time caused the intestines to become soft and mushy, with significantly reduced elasticity (Table 2). Prolonged cooking may also introduce a slight bitterness, impacting the mouthfeel negatively. This is likely because some compounds from the spices dissolve into the broth and adhere to the intestines, contributing to a bitter taste. The comprehensive sensory analysis indicated that PLI have the best sensory at 20 mins of cooking.

## CONCLUSION

This study determined the optimal cooking time for gourmet DCT by analyzing the technological characteristics of PLI. Due to the processing, which involved stir-frying followed by cooking, the moisture content in the intestines significantly decreased at 10 mins (p<0.05), with the immobile water converting into free water. As the cooking time extended, the hydrolysis of collagen and myofibrillar proteins promotes water penetration and absorption, leading to an increase in moisture content at 60 mins. Compared with RL, the fat content decreased significantly at 0 min (p<0.05), which was because the heating caused the fat cells to rupture and the fat flowed into the soup. 0–60 mins, the fat content decreased due to pre-stirring and stewing. The protein content showed no significant changes. At 20 mins, the moisture, fat, and protein content in PLI were closest to 0 min. The UFA/SUFA increased with prolonged cooking time. The main off-odor in RL was primarily attributed to cresol, indole, and 3-methylindole. The flavor compounds were most abundant when cooked for 20 mins, and no substances associated with the odor of the PLI were detected. The reason is that cooking and the addition of spices mask or eliminate unpleasant flavors. Scanning electron microscope results showed that at 20 mins, the surface of the intestines is smooth with clear pores and an intact structure, not completely destroyed. The chewiness, color, and sensory scores of PLI were optimal at 20 mins of cooking. The comprehensive analysis indicates that a cooking time of 20 mins is optimal.

The results of this study provide references for the development and utilization of pig by-products, and offer a theoretical basis for the key processing technologies and process parameters in the industrialization of DCT.

## Figures and Tables

**Table 1:** Sensory scoring sheet of cooked PLI

Scoring item	Description	Score
Appearances (20)	Good color, uniform, able to stimulate appetite	14–20
	Average color, slightly dull	7–13
	Poor color, lack of uniformity, does not stimulate appetite	0–6
Spice flavor (20)	Moderate aroma of spices, rich aroma	14–20
	Slightly light on spices, slightly bitter	7–13
	The weak aroma of spices, lacking in aroma	0–6
Large intestine flavor (20)	Moderate aroma of intestines, rich fat aroma	14–20
	Slight intestinal fishy odor, weak aroma of intestinal fat	7–13
	Strong intestinal fishy odor	0–6
Tenderness (20)	Has elasticity, good chewiness	14–20
	Moderate firmness, some chewiness	7–13
	Intestines are soft and mushy, with poor chewiness	0–6
Taste (20)	Refreshing taste, moderately salty and spicy, rich meaty aroma	14–20
	Average taste, slightly salty or slightly bland, with a faint meaty aroma	7–13
	Poor taste, too salty or too bland, lacks meaty aroma	0–6

PLI, pig large intestine.

**Table 2:** The effect of different cooking time on the texture of RL and cooked PLI

Sample^1)^	Hardness (g)	Springiness (mm)	Cohesiveness	Chewiness (g·mm)
RL	176.46±15.31^a^	1.05±0.03^a^	0.67±0.03^ab^	123.05±0.81^a^
0	86.36±2.59^d^	0.97±0.04^bc^	0.73±0.06^a^	61.16±5.09^d^
10	162.06±6.19^b^	0.93±0.01^c^	0.51±0.01^c^	76.48±3.12^c^
20	175.92±8.28^a^	1.02±0.05^ab^	0.60±0.03^b^	108.64±5.56^b^
40	150.59±7.86^b^	0.97±0.04^bc^	0.71±0.05^a^	104.78±15.60^b^
60	130.24±1.83^c^	0.79±0.02^d^	0.59±0.03^b^	60.81±1.42^d^

1) RL, raw pig large intestine; 0, pre-treated pig large intestine; 10, 20, 40 and 60 represent pre-treated pig large intestine cooked for 10 mins, 20 mins, 40 mins and 60 mins.

a–d Different letters on the same column indicate significant differences (p<0.05).

PLI, pig large intestine.

**Table 3:** The effect of different cooking time on the color of RL and cooked PLI

Sample^1)^	L*	a*	b*
RL	80.71±1.08^a^	−1.56±0.07^e^	16.14±0.54^c^
0	74.11±1.61^b^	0.79±0.31^d^	19.50±0.57^b^
10	64.45±0.30^c^	4.96±0.09^c^	24.04±0.47^a^
20	53.75±0.59^d^	6.78±0.37^a^	22.76±1.21^a^
40	44.90±2.18^e^	5.71±0.35^b^	15.90±2.52^c^
60	44.52±1.17^e^	6.08±0.06^b^	15.77±0.62^c^

1) RL, raw pig large intestine; 0, pre-treated pig large intestine; 10, 20, 40 and 60 represent pre-treated pig large intestine cooked for 10 mins, 20 mins, 40 mins and 60 mins.

a–e Different letters on the same column indicate significant differences (p<0.05).

PLI, pig large intestine.

**Table 4:** The effect of different cooking time on the moisture status of RL and cooked PLI

Sample^1)^	T_2b_ (ms)	T_21_ (ms)	T_22_ (ms)	P_2b_ (%)	P_21_ (%)	P_22_ (%)
RL	0.61±0.25^cd^	79.42±6.54^a^	1000±0.00^a^	4.25±0.05^ab^	94.76±0.14^b^	0.99±0.10^b^
0	1.15±0.00^a^	53.50±5.27^b^	921.87±15.18^b^	4.03±0.28^ab^	94.88±0.33^b^	0.88±0.04^b^
10	0.49±0.12^d^	3.37±1.43^e^	53.50±4.30^f^	3.44±1.29^b^	2.46±0.93^c^	94.11±1.24^a^
20	0.86±0.18^b^	52.25±3.73^b^	623.8±30.63^d^	4.71±0.43^a^	94.58±0.33^b^	0.71±0.20^b^
40	0.78±0.08^bc^	46.53±3.55^c^	730.31±123.03^c^	4.98±0.48^a^	94.58±0.50^b^	0.44±0.17^b^
60	0.63±0.13^cd^	37.64±0.00^d^	370.28±46.23^e^	3.75±0.35^b^	95.71±0.33^a^	0.53±0.11^b^

1) RL, raw pig large intestine; 0, pre-treated pig large intestine; 10, 20, 40 and 60 represent pre-treated pig large intestine cooked for 10 mins, 20 mins, 40 mins and 60 mins.

a–e Different letters on the same column indicate significant differences (p<0.05).

PLI, pig large intestine; T_2b_, the relaxation time of bound water; T_21_, the relaxation time of immobilized water; T_22_, the relaxation time of free water; P_2b_, The relative content of bound water; P_21_, the relative content of immobilized water; P_22_, the relative content of free water.

**Table 5:** The effect of different cooking time on the fatty acid content of RL and cooked PLI

Compound	RL	0	10	20	40	60
C10:0	0.19±0.01^a^	0.12±0.01^b^	0.12±0.01^b^	0.13±0.00^b^	0.12±0.00^b^	0.10±0.00^c^
C12:0	0.31±0.01^a^	0.12±0.00^b^	0.13±0.01^b^	0.13±0.00^b^	0.12±0.00^b^	0.10±0.00^c^
C14:0	3.23±0.09^a^	1.85±0.04^b^	1.81±0.10^b^	1.86±0.02^b^	1.81±0.01^b^	1.55±0.01^c^
C15:0	0.19±0.02^b^	0.21±0.01^a^	0.15±0.02^c^	0.15±0.00^c^	0.15±0.00^c^	0.16±0.00^c^
C16:0	22.44±1.63^ab^	23.61±0.37^a^	22.50±0.67^ab^	21.66±0.17^b^	21.59±0.23^b^	19.80±0.07^c^
C17:0	0.90±0.04^d^	1.73±0.00^a^	1.29±0.11^b^	1.12±0.01^c^	1.16±0.00^c^	1.10±0.02^c^
C18:0	22.75±0.73^c^	25.92±0.23^a^	24.69±0.64^b^	21.35±0.12^d^	22.00±0.30^cd^	19.74±0.28^e^
C20:0	0.36±0.01^c^	0.38±0.02^bc^	0.44±0.04^bc^	0.45±0.02^b^	0.46±0.01^b^	0.63±0.11^a^
C22:0	ND	ND	0.43±0.04^d^	0.75±0.04^b^	0.62±0.06^c^	1.10±0.01^a^
C16:1	2.81±0.48^a^	1.98±0.01^b^	1.57±0.09^c^	2.21±0.02^b^	1.59±0.00^b^	1.49±0.02^c^
C20:1	1.59±0.09^a^	1.42±0.05^bc^	1.37±0.09^bc^	1.46±0.03^b^	1.58±0.00^a^	1.31±0.05^c^
C18:1n9c	28.78±3.79^c^	35.37±0.27^a^	34.21±0.59^ab^	36.17±0.45^a^	35.50±0.16^a^	32.40±0.01^b^
C18:2n6c	14.55±0.85^b^	5.27±0.03^e^	9.49±0.10^d^	10.08±0.08^cd^	10.39±0.04^c^	16.71±0.23^a^
C18:3n3	0.99±0.09^b^	0.23±0.05^c^	0.88±0.12^b^	1.37±0.60^b^	1.44±0.59^b^	2.28±0.10^a^
C20:3n6	0.22±0.00^c^	0.24±0.00^b^	0.21±0.00^c^	0.18±0.01^d^	0.16±0.00^e^	0.30±0.01^a^
C20:4n6	0.70±0.04^d^	1.56±0.03^a^	0.70±0.07^d^	0.95±0.13^c^	0.94±0.05^c^	1.21±0.04^b^
SFA	50.37±2.43^b^	53.94±0.33^a^	51.56±0.28^b^	47.58±0.17^c^	48.04±0.50^c^	44.29±0.26^d^
MUFA	33.18±3.25^d^	38.77±0.24^ab^	37.16±0.41^bc^	39.84±0.45^a^	39.04±0.16^ab^	35.21±0.06^cd^
PUFA	16.45±0.84^b^	7.30±0.10^e^	11.28±0.16^d^	12.57±0.59^c^	12.93±0.66^c^	20.49±0.30^a^
UFA	49.63±2.43^c^	46.06±0.33^d^	48.44±0.28^c^	52.42±0.17^b^	51.96±0.50^b^	55.71±0.26^a^
UFA/SFA	0.99±0.09^c^	0.85±0.01^d^	0.94±0.10^c^	1.10±0.01^b^	1.08±0.02^b^	1.26±0.01^a^

a–e Different letters on the same row indicate significant differences (p<0.05).

RL, raw pig large intestine; 0, pre-treated pig large intestine; 10, 20, 40 and 60 represent pre-treated pig large intestine cooked for 10 mins, 20 mins, 40 mins and 60 mins.

ND indicates that the substance was not detected.

PLI, pig large intestine; SFA, saturated fatty acids; MUFA, monounsaturated fatty acids; PUFA, polyunsaturated fatty acids; UFA, unsaturated fatty acids.

**Table 6:** The effect of different cooking time on the odor activity values of RL and cooked PLI

No.	Compound	Threshold (mg/kg)	RL	0	10	20	40	60
1	3-Heptanone	0.08	139.75	185.38	76.75	163.63	116.00	132.63
3	5-Nonanone	0.0082	1,401.22	1,759.76	754.88	2,131.71	1,530.49	1,336.59
4	4’-Methylacetophenone	2.1	1.44	1.45	0.76	1.12	0.89	0.65
8	6-Undecanone	0.085	ND	ND	ND	ND	9.88	31.18
11	Benzaldehyde	0.75089	ND	ND	2,627.27	4,527.27	ND	ND
12	Nonanal	0.0011	ND	1,236.36	ND	ND	ND	ND
15	4-Methoxybenzaldehyde	0.047	ND	160.43	297.02	329.79	220.00	190.64
16	2,4-decadienal	0.0003	ND	ND	3,300.00	ND	ND	ND
17	(E, E)-2,4-decadienal	0.00008	ND	ND	ND	ND	10,375.0	25,375.0
19	2,3-dimethylpentan-2-ol	1.6	11.49	5.51	2.77	7.51	6.63	6.85
20	Eucalyptol	0.0011	ND	ND	1,427.27	2,527.27	ND	3,954.55
21	Linalool	0.00022	ND	ND	19,090.91	29,500.00	16,909.09	40,454.55
22	Phenylethanol	0.56423	3.35	3.19	2.13	2.46	2.00	1.45
23	4-Terpinenol	1.2	ND	ND	0.62	0.81	0.00	1.49
24	α-Terpineol	1.2	ND	ND	1.07	0.67	1.59	1.04
26	Cinnamyl Alcohol	0.077	ND	ND	87.92	ND	ND	ND
28	p-Cresol	0.01	1,087.00	ND	315.00	ND	ND	ND
29	Eugenol	0.0025	128.00	304.00	984.00	2,348.00	1,436.00	2,600.00
32	Anethole	0.015	274.67	1,996.00	1,603.33	2,720.67	2,244.00	4,419.33
33	Estragole	0.016	ND	87.50	36.88	346.25	ND	46.88
34	Methyl benzoate	0.073	23.97	21.23	0.00	13.01	10.00	0.00
35	Linalyl acetate	1	ND	ND	ND	ND	2.43	8.25
36	α-Terpineol acetate	2.5	ND	ND	0.31	0.35	0.20	0.46
37	Ethyl decanoate	0.005	66.00	268.00	0.00	478.00	106.00	88.00
39	Methyl palmitate	2	ND	0.20	0.00	0.28	0.17	0.00
41	5-Decalactone	0.066	ND	9.70	ND	ND	ND	ND
45	Phellandrene	0.04	ND	ND	ND	9.75	ND	44.75
48	Mesitylene	0.7	4.33	27.07	2.47	6.01	7.49	4.21
49	3-Ethyltoluene	0.8	ND	ND	ND	1.66	ND	ND
52	Indole	0.04	196.25	106.25	64.75	ND	82.25	ND
55	3-Methylindole	0.00041	4,414.63	ND	ND	ND	3829.27	ND

RL, raw pig large intestine; 0, pre-treated pig large intestine; 10, 20, 40 and 60 represent pre-treated pig large intestine cooked for 10 mins, 20 mins, 40 mins and 60 mins.

ND indicates that the substance was not detected.

PLI, pig large intestine.

## SUPPLEMENTARY MATERIAL

**Supplement 1**. The effect of different cooking time on volatile flavor compounds of RL and cooked PLI.
