# Supplementary material for: Exploitation of pig by-product: physicochemical properties and flavor of Dachang Tang: effect of cooking time
Source: Anim Biosci. 2025 Apr 28;38(10):2266–79. doi: 10.5713/ab.24.0894 (PMC12415379; doi:10.5713/ab.24.0894)
Supplement: Supplementary file 1 [file ab-24-0894-Supplementary-1.pdf]

Supplement 1. The effect of different cooking time on volatile flavor compounds of RL and cooked PLI

No.	Compound	CAS	Content（mg/kg）						Flavor description
			RL	0	10	20	40	60	
	Keton								
1	3-Heptanone	106-35-4	11.18±0.96^{ab}	14.83±4.14^{a}	6.14±0.36^{c}	13.09±2.47^{ab}	9.28±2.59^{bc}	10.61±0.22^{ab}	green
2	2,4-Dimethyl-3-heptanone	18641-71-9	11.49±1.97^{b}	14.43±0.97^{ab}	6.19±0.75^{c}	17.48±2.67^{a}	12.55±2.99^{b}	10.96±4.72^{ab}	
3	5-Nonanone	502-56-7	5.09±0.25^{b}	12.37±3.19^{a}	4.14±0.72^{b}	9.64±3.14^{a}	8.76±1.11^{a}	13.44±2.71^{a}	
4	4'-Methylacetophenone	122-00-9	3.02±0.25^{a}	3.04±0.52^{a}	1.60±0.22^{c}	2.36±0.46^{b}	1.87±0.33^{bc}	1.37±0.23^{c}	floral fatty
5	Butyrophenone	495-40-9	7.46±0.37	3.67±0.42	ND	ND	ND	ND	camphoreous
6	4-Methylpropiophenone	5337-93-9	5.62±0.59^{a}	ND	ND	4.21±1.90^{ab}	2.46±0.23^{c}	3.14±1.17^{bc}	
7	1-Phenyl-2-pentanone	6683-92-7	ND	ND	ND	ND	0.58±0.03	0.53±0.15	

8	6-Undecanone	927-49-1	3.26±0.76[a]	ND	1.84±0.40[bc]	ND	0.84±0.15[c]	2.65±0.44[ab]	
9	2-Pentadecanone	2345-28-0	ND	0.66±0.08[a]	ND	0.45±0.08[ab]	0.50±0.17[ab]	0.36±0.03[b]	floral fatty
10	7-Tridecanone	462-18-0	0.42±0.04	ND	ND	ND	ND	ND	
		合计	46.52±3.92[a]	48.78±9.37[a]	19.30±2.52[b]	47.23±8.28[a]	36.85±7.50[a]	41.56±3.44[a]	
	Aldehyde								
11	Benzaldehyde	100-52-7	ND	ND	2.89±0.16	4.98±0.90	ND	ND	fruity almond cherry
12	Nonanal	124-19-6	ND	1.36±0.15	ND	ND	ND	ND	fatty orange
13	(Z)-Cinnamaldehyde	57194-69-1	ND	33.89±2.73[a]	10.11±1.92[c]	34.21±3.90[a]	17.67±0.75[b]	11.23±1.60[c]	spicy cinnamon
14	2-Methoxybenzaldehyde	135-02-4	ND	0.71±0.25	ND	0.34±0.01	ND	ND	anisic hawthorn almond
15	4-	123-11-5	ND	7.54±2.93[b]	13.96±0.05[a]	15.50±1.21[a]	10.34±1.47[b]	8.96±0.80[b]	Anisic woody

	Methoxybenzaldehyde								creamy spicy
16	2,4-decadienal	2363-88-4	ND	ND	0.99±0.09	ND	ND	ND	fat oily green
17	(E, E)-2,4-decadienal	25152-84-5	ND	ND	ND	ND	0.83±0.09	2.03±0.70	fatty oily waxy
18	2-Undecenal	2463-77-6	ND	1.84±0.23^{ab}	ND	1.03±0.07^{ab}	0.80±0.23^{a}	0.83±0.22^{b}	fruity fresh orange
Total				44.72±6.10^{b}	27.61±2.13^{c}	55.46±5.28^{a}	29.19±2.82^{c}	23.05±2.59^{c}	
	Alcohol								
19	2, 3-dimethylpentan-2-ol	4911-70-0	18.39±1.03^{a}	8.82±1.50^{b}	4.43±1.09^{c}	12.01±4.67^{b}	10.60±1.89^{b}	10.96±1.88^{b}	
20	Eucalyptol	470-82-6	ND	ND	1.57±0.27^{b}	2.78±0.91^{ab}	ND	4.35±0.99^{a}	herbal
21	Linalool	78-70-6	ND	ND	4.20±1.27^{bc}	6.49±1.13^{b}	3.72±0.46^{c}	8.90±1.76^{a}	floral orange waxy rosy
22	Phenylethanol	60-12-8	1.89±0.17^{a}	1.80±0.19^{a}	1.20±0.14^{a}	1.39±0.17^{b}	1.13±0.10^{b}	0.82±0.11^{c}	floral rosy honey

23	4-Terpinenol	562-74-3	ND	ND	0.74±0.04[b]	0.97±0.22[b]	ND	1.79±0.47[a]	spicy woody mentholic citrus
24	α-Terpineol	98-55-5	ND	ND	1.28±0.19[b]	0.80±0.06[b]	1.91±0.46[a]	1.25±0.36[b]	woody lilac citrus floral
25	3-Phenyl-1-propanol	122-97-4	ND	ND	0.33±0.05	ND	ND	ND	spicy cinnamon fruity floral honey balsamic
26	Cinnamyl Alcohol	104-54-1	ND	ND	6.77±0.60	ND	ND	ND	cinnamon spicy floral green balsamic
27	1,12-Dodecanediol	5675-51-4	ND	0.40±0.04	ND	ND	ND	ND	
Total			19.65±0.40[bc]	10.89±1.82[d]	21.33±2.23b[c]	24.44±6.56[ab]	17.34±2.73[c]	28.05±2.50[a]	

	Phenols								
28	*p*-Cresol	106-44-5	10.87±1.41	ND	3.15±0.44	ND	ND	ND	phenolic narcissus animal
29	Eugenol	97-53-0	0.32±0.04d	0.76±0.20^{cd}	2.46±0.21^{bc}	5.87±1.35^{a}	3.59±0.57^{b}	6.50±1.58^{a}	spicy clove
Total			11.18±1.40^{a}	0.76±0.20^{d}	5.61±0.36^{bc}	5.87±1.35^{b}	3.59±0.57^{c}	6.50±1.58^{b}	
	Ethers								
30	Methyl allyl trisulfide	34135-85-8	ND	ND	0.40±0.06	ND	ND	ND	alliaceous creamy garlic onion
31	(Ethoxymethyl)benzene	539-30-0	ND	0.55±0.18	ND	ND	ND	ND	fruity
32	Anethole	104-46-1	4.12±1.19^{e}	29.94±6.31^{cd}	24.05±1.32^{d}	40.81±4.98^{b}	33.66±2.08^{bc}	66.29±6.31^{a}	anise
33	Estragole	140-67-0	ND	1.40±0.20^{b}	0.59±0.02^{c}	5.54±0.11^{a}	ND	0.75±0.12^{c}	anisic green herbal

Total			4.12±1.19[e]	31.90±6.29[cd]	25.04±1.36[d]	46.38±4.88[b]	33.66±2.08[c]	67.04±6.35[a]	
	Esters								
34	Methyl benzoate	93-58-3	1.75±0.44[a]	1.55±0.37[ab]	ND	0.95±0.29[bc]	0.73±0.24[c]	ND	cherry
35	Linalyl acetate	115-95-7	ND	ND	ND	ND	2.43±0.83	8.25±0.34	
36	α-Terpineol acetate	80-26-2	ND	ND	0.77±0.07[bc]	0.87±0.12[ab]	0.49±0.08[c]	1.16±0.34[a]	herbal citrus spicy woody floral waxy
37	Ethyl decanoate	110-38-3	0.33±0.05[b]	1.34±0.61[ab]	ND	2.39±1.57[a]	0.53±0.15[b]	0.44±0.12[b]	waxy fruity
38	Eugenol acetate	93-28-7	ND	1.54±0.12[a]	ND	0.55±0.11[b]	0.35±0.07[b]	0.43±0.11[b]	spicy fresh woody clove
39	Methyl palmitate	112-39-0	ND	0.39±0.05[b]	ND	0.55±0.04[a]	0.33±0.02[b]	ND	waxy oily fatty
40	Methyl oleate	112-62-9	ND	ND	ND	0.69±0.22	ND	ND	fatty
41	5-Decalactone	705-86-2	ND	0.64±0.01	ND	ND	ND	ND	coconut creamy

									fatty
42	Methyl petroselinate	2777-58-4	ND	ND	ND	ND	0.82±0.30	0.50±0.06	
Total			2.09±0.49[cd]	4.61±1.15[bc]	0.77±0.07[d]	5.82±2.42[b]	5.38±0.20[b]	10.80±2.41[a]	
	Acid								
43	Glycine	7501-44-2	0	0.63±0.19	0	0	0	0	
	Hydrocarbon								
44	Hexadecanal	629-80-1	ND	ND	0.42±0.06[b]	0.69±0.09[a]	0.67±0.07[a]	0.81±0.17[a]	
45	Phellandrene	99-83-2	ND	ND	ND	0.39±0.03	ND	1.79±0.12	
46	Butane	106-97-8	9.49±0.78	ND	ND	ND	ND	ND	
47	Hexylbenzene	1077-16-3	0.56±0.11	ND	ND	ND	ND	ND	
Total			10.05±0.87[a]	0	0.41±0.08[b]	0.95±0.20[b]	0.67±0.07[b]	1.12±0.15[b]	
	Aromatic								

48	Mesitylene	108-67-8	3.03±0.48[d]	18.95±0.45[a]	1.73±0.16[e]	4.21±0.822[c]	5.24±0.40[b]	2.95±0.38[d]	
49	3-Ethyltoluene	620-14-4	ND	ND	ND	1.33±0.64	ND	ND	
50	Isobutyrophenone	611-70-1	2.68±0.28[ab]	2.94±0.89[a]	1.94±0.20[b]	2.54±0.48[ab]	2.44±0.21[ab]	2.10±0.13[ab]	green
51	Caprophenone	942-92-7	0.85±0.02[a]	0.56±0.09[b]	0.40±0.06[b]	0.52±0.03[b]	0.53±0.09[b]	0.48±0.17[b]	
Total			6.56±0.76[cd]	22.27±1.16[a]	4.07±0.25[e]	8.60±1.83[b]	8.20±0.24[bc]	5.52±0.56[de]	
	Others								
52	Indole	120-72-9	7.85±0.24[a]	4.25±1.81[b]	2.59±0.17[b]	ND	3.29±0.62[b]	ND	fecal
53	7-Methylindole	933-67-5	ND	ND	1.51±0.15	ND	ND	ND	
54	2-Methylindole	95-20-5	ND	1.23±0.02	ND	ND	ND	ND	
55	3-Methylindole	83-34-1	1.81±0.05	ND	ND	ND	1.57±0.10	ND	fecal
56	Valeric anhydride	2082-59-9	0.71±0.09	ND	ND	ND	ND	ND	
Total			10.36±0.25	5.07±1.44	4.11±0.31	0	4.86±0.67	0	

Total	111.54±5.21^{b}	169.37±26.28^{a}	108.26±3.04^{c}	194.73±18.84^{a}	140.19±14.92^{b}	183.63±12.95^{a}

PLI, pig large intestine; RL, raw pig large intestine

Different letters on the same column indicate significant differences ($p<0.05$).

ND indicates that the substance was not detected.

The odor perception referred from http://www.perflavory.com/index.html.

6
